# Predictive value of the uric acid-to-albumin ratio for contrast-induced nephropathy after percutaneous coronary intervention and construction of a clinical prediction model in patients with coronary artery disease

**DOI:** 10.3389/fcvm.2026.1788251

**Published:** 2026-05-21

**Authors:** Yuxin Zhu, Qian Liu, Yun Deng, Jinfeng Chen, Yunxian Chen, Baofeng Chen

**Affiliations:** 1 Department of Cardiology, Yuebei People’s Hospital, Shaoguan, Guangdong, China; 2 First School of Clinical Medicine, Guangdong Medical University, Zhanjiang, Guangdong, China; 3 Department of Ultrasound Diagnostics, Yuebei People’s Hospital, Shaoguan, Guangdong, China; 4 Department of Cardiology, YueBei People’s Hospital Joint Postgraduate Training Base, Guangdong Medical University, Shaoguan, Guangdong, China

**Keywords:** contrast-induced nephropathy, coronary heart disease, nomogram, percutaneous coronary intervention, uric acid-to-albumin ratio

## Abstract

**Background:**

Percutaneous coronary intervention (PCI) has become the cornerstone of coronary revascularization, and contrast-induced nephropathy (CIN) is one of the most frequent serious complications of PCI and is associated with prolonged hospitalization and higher mortality. We hypothesized that the uric acid-to-albumin ratio (UAR), a readily obtainable inflammatory-metabolic index, could enhance pre-procedural risk stratification for CIN.

**Methods:**

A consecutive cohort of 788 Chinese patients with coronary artery disease scheduled for elective PCI was enrolled. CIN was defined as an absolute serum creatinine rise ≥ 44.2 µmol/L or a ≥25% increase from baseline within 72 h of contrast exposure. Independent predictors were identified by multivariable logistic regression, and a base model was compared with a UAR-augmented model using the area under the ROC curve, net reclassification index, and integrated discrimination improvement. A nomogram was constructed, and a decision-curve analysis was performed.

**Results:**

CIN occurred in 76 patients (9.6%). UAR was the strongest single discriminator (AUC: 0.790) and remained independently associated with CIN (OR = 1.64; 95% CI: 1.42–1.90). Adding UAR to the base model increased the AUC from 0.850 to 0.921 (*P* < 0.001), yielded a categorical NRI of 0.296 and an IDI of 0.120 (both *P* < 0.001). Finally, we constructed a nomogram incorporating UAR as a predictor, and both the calibration plot and the clinical decision curve confirmed the model's accuracy and substantial net benefit.

**Conclusion:**

UAR is a novel predictor of CIN after elective PCI. Its incorporation into a simple five-item nomogram significantly improves pre-procedural risk estimation and clinical decision-making. External validation is warranted before widespread implementation.

## Introduction

With the global incidence of coronary artery disease continuing to climb, percutaneous coronary intervention (PCI) has become the cornerstone of myocardial revascularization (1–3). Contrast-induced nephropathy (CIN) is one of the common complications of PCI and has multiple definitions (4). The European Society of Urogenital Radiology defines it as an increase in serum creatinine levels by ≥0.5 mg/dL within 48–72 h after contrast administration, representing a rise of >25% from the baseline value (4, 5). The Kidney Disease Improvement and Outcomes (KDIGO) Initiative defines it as an increase in serum creatinine levels by ≥0.3 mg/dL (26.5 μmol/L) within 48 h, or a rise of >1.5–1.9 times the baseline value within 7 days, or a urine output of <0.5 mL/kg/h after contrast administration (6). The incidence of CIN varies significantly depending on the patients' baseline risk characteristics, in patients undergoing elective PCI with preserved renal function and no major comorbidities, the risk of CIN is very low (<3%), in unselected populations, pooled estimates from meta-analyses range from approximately 13.3 to 14.4%, in patients with acute myocardial infarction (AMI), the incidence increases substantially to 10%–30%, and may exceed 50% when complicated by cardiogenic shock (7–15). CIN leads to increased mortality rates, length of hospital stay, and hospitalization costs (16, 17). Furthermore, CIN is the third leading cause of acute renal failure (ARF) (after acute renal hypoperfusion and drug-induced nephrotoxicity), accounting for 11% of such ARF cases, as it can lead to significant impairment of renal function and, in severe cases, necessitate dialysis (18, 19). Established risk factors include advanced age, hypertension, diabetes mellitus (DM), chronic kidney disease (CKD), heart failure, peripheral vascular disease, anaemia, malnutrition, contrast volume, and osmolality. Furthermore, a (V/CrCl) ratio > 3.7 has been demonstrated to be an independent and significant predictor of early abnormal elevation of serum creatinine following PCI (20, 21). The Mehran score integrates eight variables (hypotension, intra-aortic balloon pump, congestive heart failure, chronic kidney disease, diabetes, age > 75 years, anaemia and contrast volume) and remains the most widely cited model for predicting contrast-induced nephropathy (22). However, its inclusion of per-procedural elements limits utility for pre-operative risk stratification (23). Current strategies for CIN prevention include periprocedural hydration as the cornerstone, discontinuation of nephrotoxic medications such as diuretics 24–48 h before elective PCI, and periprocedural statin therapy (24–28). The efficacy of N-acetylcysteine and sodium bicarbonate remains debated, with meta-analyses showing inconsistent results (29–32). Because no single preventive strategy has proven universally effective, early risk stratification remains essential to identify high-risk.

Uric acid is the final metabolite of purine catabolism in humans; elevated serum levels are associated with impaired endothelial function, heightened inflammatory responses, and augmented oxidative stress (33). Previous studies have demonstrated that uric acid is significantly increased in patients who develop CIN (34). Serum albumin, the most abundant protein in the circulation, exerts anti-inflammatory, anti-apoptotic, and antioxidant effects, and has been identified as an independent predictor of CIN (35, 36). In recent years, the Uric Acid to Albumin Ratio (UAR), as a new inflammatory-metabolic marker, has shown unique value in predicting the prognosis of cardiovascular diseases. Studies have shown that UAR exhibits significant predictive ability in various cardiovascular diseases. In patients with ST-segment elevation myocardial infarction (STEMI), UAR has been identified as an independent predictor of all-cause mortality and cardiovascular mortality, with superior predictive performance compared to uric acid or albumin levels alone (37). Additionally, UAR has been used to predict long-term mortality in patients after acute type A aortic dissection surgery (38). In patients with non-ST-segment elevation myocardial infarction (NSTEMI), UAR is used to assess the severity of coronary artery disease, and its predictive ability is superior to other inflammatory markers (39). Furthermore, UAR has shown significant predictive value in forecasting all-cause and cardiovascular mortality in diabetic patients, further confirming its broad applicability across different populations (40). Nevertheless, whether the UAR is linked to CIN after PCI in coronary artery disease remains undefined.

Using a Chinese cohort of patients with coronary artery disease undergoing PCI, this study investigated the association between the UAR and incident CIN and developed a risk-prediction model, thereby providing an early warning tool and potential preventive strategies to improve quality of life and clinical outcomes in this high-risk population.

## Method

### Participants and selection

**Figure 1:**
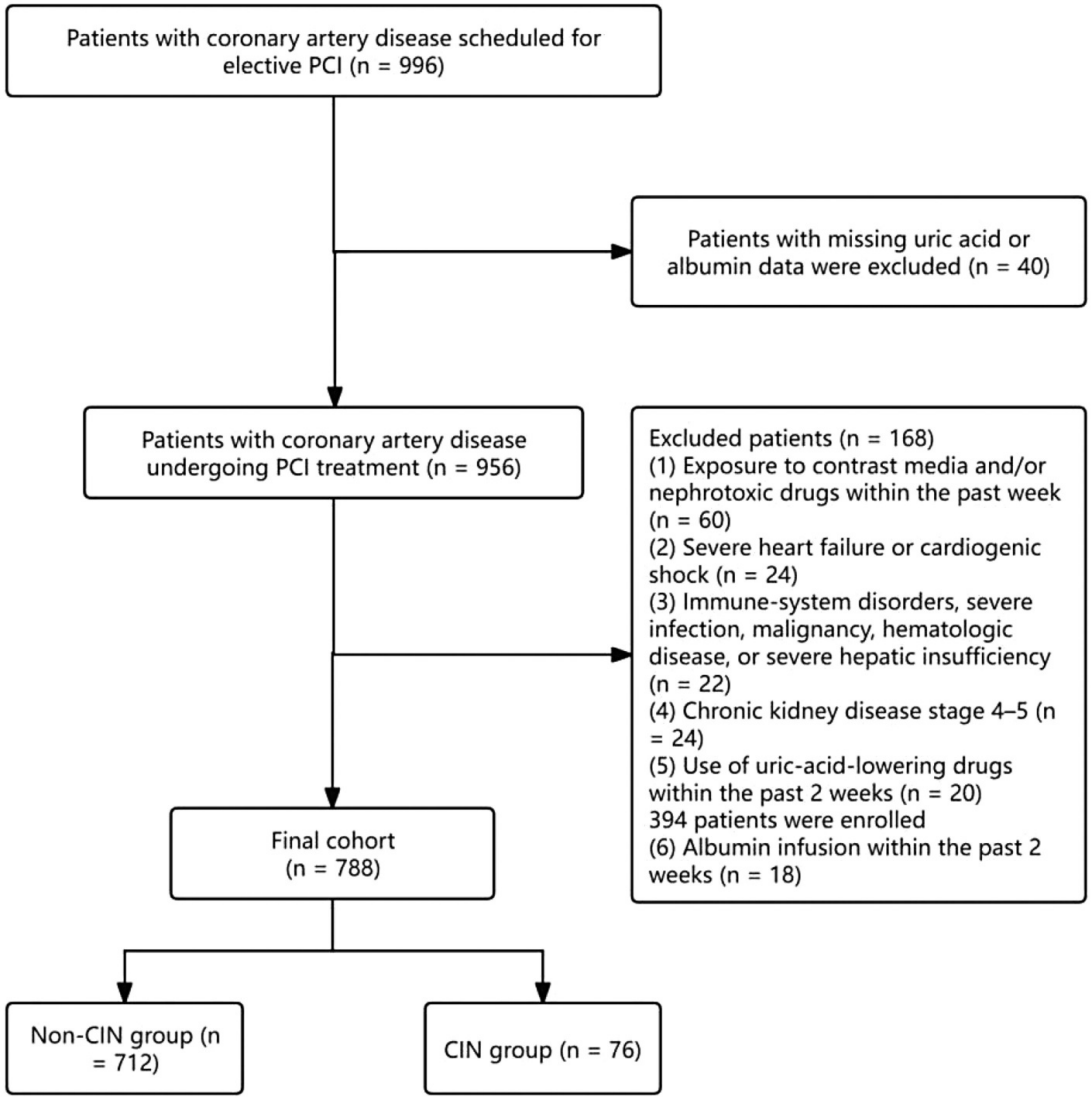
Flowchart of patient inclusion.

This study enrolled a total of 788 patients with coronary heart disease who underwent PCI at the Department of Cardiology, Yuebei People's Hospital between February 2023 and October 2024. The inclusion criteria were: (1) age ≥ 18 years; (2) diagnosis of chronic coronary syndrome requiring PCI; and (3) availability of complete medical records. Patients were excluded if they met any of the following criteria: (1) exposure to contrast agents and/or drugs affecting renal function within the past week; (2) severe heart failure or cardiogenic shock; (3) presence of immune system diseases, severe infections, malignant tumors, hematological diseases, or severe liver dysfunction; (4) chronic kidney disease stage 4–5; (5) use of uric acid-lowering drugs (such as allopurinol, febuxostat, benzbromarone) within the past 2 weeks; or (6) receipt of albumin infusion therapy within the past 2 weeks (Figure 1).

All PCI procedures were performed by experienced interventional cardiologists following established operational standards, using standardized guidewires, balloons, and stent devices. Throughout the surgical procedure, ioversol injection (concentration 350 mgI/mL, manufactured by Jiangsu Hengrui Medicine Co., Ltd.) was uniformly administered as the contrast agent, with the specific dosage determined by the operating physician based on individual patient conditions. The collection of clinical data for this study complied with medical ethics standards and received approval from the hospital ethics committee (Ethics No.: YBSKY-2024-149-001).

### Clinical data

General data of the enrolled patients were collected, including age, gender, smoking history, admission heart rate, hypertension, diabetes mellitus (DM), etc. Preoperative routine laboratory test results, such as blood routine, fasting blood glucose, total cholesterol (TC), triglyceride (TG), low-density lipoprotein cholesterol (LDL-C), high-density lipoprotein cholesterol (HDL-C), uric acid, estimated glomerular filtration rate (eGFR), albumin, left ventricular ejection fraction (LVEF), and medication status [diuretics, angiotensin-converting enzyme inhibitor (ACEI)/angiotensin II receptor blocker (ARB) drugs] and other indicators were also collected.

### Exposure and outcome

According to international guidelines, CIN is defined as an absolute increase in serum creatinine ≥ 44.2 μmol/L or a relative increase ≥ 25% from baseline within 72 h of contrast exposure, excluding other renal injury factors (16). The exposure factor is UAR.

### Statistical analysis

Measurement data following a normal distribution were expressed as mean ± standard deviation, and independent samples *t*-test was used to compare data between the two groups; measurement data not conforming to normal distribution were expressed as median (interquartile range), and Mann–Whitney *U*-test was used for comparison between the two groups; Count data were presented as cases (constituent ratio), and *χ*^2^ test was used to compare differences between groups.

Variables that exhibited significant differences were entered into a univariate logistic regression analysis. Variables with *P* < 0.05 were then selected and incorporated into a multivariate logistic regression model to identify independent correlates. Finally, receiver-operating characteristic (ROC) curves were constructed to evaluate the predictive value of these independent indicators for CIN. Based on the results of multivariate logistic regression analysis, a base model was established to predict coronary heart disease patients undergoing PCI treatment. By calculating net reclassification improvement (NRI), integrated discrimination improvement (IDI), and area under the curve (AUC) values, the predictive incremental effect of UAR on CAD patients undergoing PCI treatment was compared. Finally, we constructed a nomogram including UAR risk factors to predict the risk of CIN in coronary heart disease patients undergoing PCI treatment, and used calibration curves and clinical decision curves to evaluate the performance of the model.

All statistical analyses were performed using R software (version 4.3.3), and a two-tailed *P* < 0.05 was considered statistically significant.

## Results

### Baseline characteristics

Among 788 patients, 76 cases (approximately 9.64%) developed CIN (Table 1). Comparison of baseline data between the two groups showed no statistical significance in age, gender, hypertension, platelets, glucose, TC, LDL-C, HDL-C, surgical duration, and ACEI/ARB drugs (*P* > 0.05); patients in the CIN group had higher admission heart rate, DM, uric acid, UAR, and diuretic usage rate than the non-CIN group, while TG, hemoglobin, lymphocytes, eGFR, albumin, and LVEF were lower than those in the non-CIN group, with statistically significant differences (*P* < 0.05).

**Table 1:** Comparison of characteristics between CIN and non-CIN groups in patients with coronary heart disease after PCI.

Characteristics	Total (*n* = 788)	Non-CIN (*n* = 712)	CIN (*n* = 76)	*P*
Age, years	65.25 ± 10.23	65.04 ± 10.00	67.21 ± 12.03	0.132
Male, *n* (%)	574 (72.84)	524 (73.60)	50 (65.79)	0.187
Smoking, *n* (%)	182 (23.10)	162 (22.75)	20 (26.31)	0.577
Heart rate, beats/minute	80.53 ± 16.02	79.57 ± 15.65	89.53 ± 16.71	<.001
Hemoglobin, g/L	133.89 ± 19.96	134.79 ± 19.39	125.45 ± 23.15	<.001
WBC, 10^9^/L	10.76 ± 5.30	10.92 ± 5.58	9.27 ± 3.13	0.435
Platelet, 10^9^/L	237.41 ± 65.35	235.62 ± 63.28	254.18 ± 80.87	0.056
Lymphocyte, 10^9^/L	1.81 ± 0.88	1.85 ± 0.89	1.50 ± 0.72	<.001
Glucose, mmol/L	7.22 ± 3.71	7.23 ± 3.75	7.07 ± 3.37	0.700
TC, mmol/L	5.78 ± 1.71	4.91 ± 1.30	4.87 ± 1.47	0.157
TG, mmol/L	1.76 ± 1.66	1.79 ± 1.71	1.48 ± 1.14	0.037
LDL-C, mmol/L	2.61 ± 1.26	2.61 ± 1.25	2.64 ± 1.36	0.853
HDL-C, mmol/L	1.75 ± 0.47	1.77 ± 0.49	1.54 ± 0.98	0.278
Uric acid, μmol/L	389.54 ± 99.62	381.83 ± 95.29	461.77 ± 110.43	<.001
eGFR, mL/min/1.73 m^2^	78.40 ± 19.12	79.20 ± 18.22	70.83 ± 25.00	0.006
Albumin, g/L	41.13 ± 4.00	41.50 ± 3.75	37.69 ± 4.60	<.001
UAR	9.55 ± 2.60	9.25 ± 2.32	12.41 ± 3.33	<.001
LVEF, %	55.53 ± 9.43	56.45 ± 8.67	46.92 ± 11.75	<.001
DM, *n* (%)	282 (35.79)	244 (34.27)	38 (50)	<.001
Hypertension, *n* (%)	496 (62.94)	440 (61.80)	56 (73.68)	0.056
Contrast dosage, mL	70.03 ± 16.33	69.46 ± 14.93	75.42 ± 25.56	0.049
Surgical duration, min	31.47 ± 17.75	30.90 ± 16.33	36.87 ± 27.31	0.065
Diuretic, *n* (%)	300 (38.07)	238 (33.43)	62 (81.58)	<.001
ACEI/ARB, *n* (%)	642 (81.47)	582 (81.74)	60 (78.95)	0.659

WBC, white blood cell; TC, total cholesterol; TG, triglyceride; LDL-C, low-density lipoprotein cholesterol; HDL-C, high-density lipoprotein cholesterol; UAR, uric acid-to-albumin ratio; DM, diabetes mellitus; LVEF, left ventricular ejection fraction; ACEI, angiotensin-converting enzyme inhibitor; ARB, angiotensin Ⅱ receptor blocker; PCI, percutaneous coronary intervention; CIN, contrast-induced nephropathy; eGFR, estimated glomerular filtration rate.

### Univariate and multivariate logistic regression analysis

Univariate logistic regression analysis showed that multiple indicators were significantly correlated with the study subjects (*P* < 0.05) (Table 2). Among them, UAR (OR = 1.60, 95% CI: 1.43–1.79), DM (OR = 1.92, 95% CI: 1.19–3.09), diuretic use (OR = 8.82, 95% CI: 4.84–16.08), heart rate (OR = 1.03, 95% CI: 1.02–1.05) and contrast agent dosage (OR = 1.02, 95% CI: 1.01–1.03) were risk factors. Hemoglobin (OR = 0.98), lymphocyte count (OR = 0.52), LVEF (OR = 0.92), and eGFR (OR = 0.98) were protective factors. TG (*P* = 0.121) was not statistically significant.

**Table 2:** Feature selection results of univariate and multivariate analyses.

Factors	Univariate analysis		Multivariate analysis	
	OR (95% CI)	*P*	OR (95% CI)	*P*
UAR	1.60 (1.43, 1.79)	<.001	1.64 (1.42, 1.90)	<.001
DM	1.92 (1.19, 3.09)	0.007	2.22 (1.19, 4.16)	0.012
Diuretic	8.82 (4.84, 16.08)	<.001	9.01 (4.37, 18.56)	<.001
Heart rate	1.03 (1.02, 1.05)	<.001	1.03 (1.01, 1.04)	0.006
Hemoglobin	0.98 (0.97, 0.99)	<.001	0.98 (0.97, 1.00)	0.137
Lymphocyte	0.52 (0.36, 0.76)	<.001	0.69 (0.43, 1.08)	0.105
TG	0.83 (0.66, 1.05)	0.121	–	–
Contrast dosage	1.02 (1.01, 1.03)	0.004	1.01 (1.00, 1.03)	0.142
LVEF	0.92 (0.90, 0.94)	<.001	0.95 (0.92, 0.98)	<.001
eGFR	0.98 (0.97, 0.99)	<.001	1.01 (1.00, 1.03)	0.110

OR, odds ratio; CI, confidence interval; UAR, uric acid-to-albumin ratio; DM, diabetes mellitus; TG, triglyceride; LVEF, left ventricular ejection fraction; eGFR, estimated glomerular filtration rate.

Multivariate logistic regression analysis identified several independent predictors of the outcome (Table 2). The UAR was significantly associated with an increased risk (OR = 1.64, 95% CI: 1.42–1.90). DM also showed a positive correlation (OR = 2.22, 95% CI: 1.19–4.16), while diuretic use demonstrated the strongest association (OR = 9.01, 95% CI: 4.37–18.56). Higher heart rate was linked to an elevated risk (OR = 1.03, 95% CI: 1.01–1.04). Conversely, LVEF was inversely associated with the outcome (OR = 0.95, 95% CI: 0.92–0.98).

### Comparative discrimination of independent predictors for CIN

**Figure 2:**
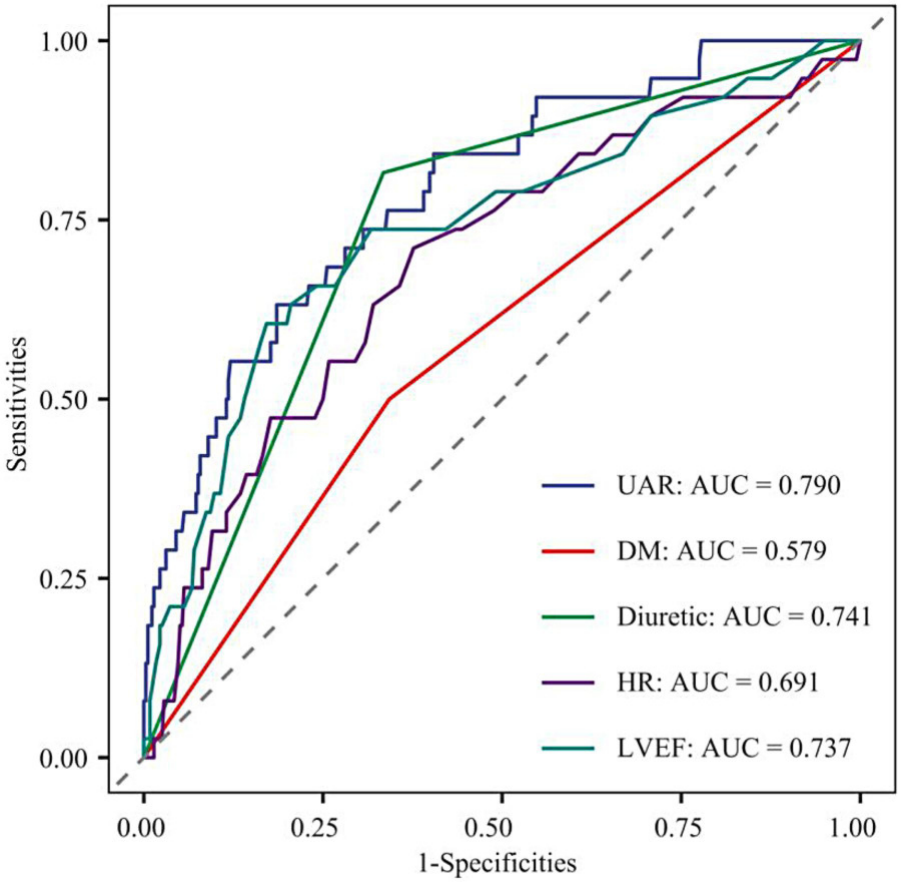
ROC curves of individual markers for predicting CIN after PCI in patients with coronary artery disease.

To evaluate the predictive efficacy of independent risk factors for CIN, ROC curve analysis was performed, with the AUC as the primary metric (Figure 2).

ROC curve analysis showed that the UAR had the highest predictive efficacy for CIN (AUC = 0.790). Diuretic use (AUC = 0.741) and LVEF (AUC = 0.737) also exhibited good predictive value. Heart rate had moderate predictive ability (AUC = 0.691), while DM had a discriminative ability close to random level (AUC = 0.579). The AUC values of the factors were ranked as UAR > diuretic use > LVEF > heart rate > DM.

### Predictive value of UAR for CIN

**Figure 3:**
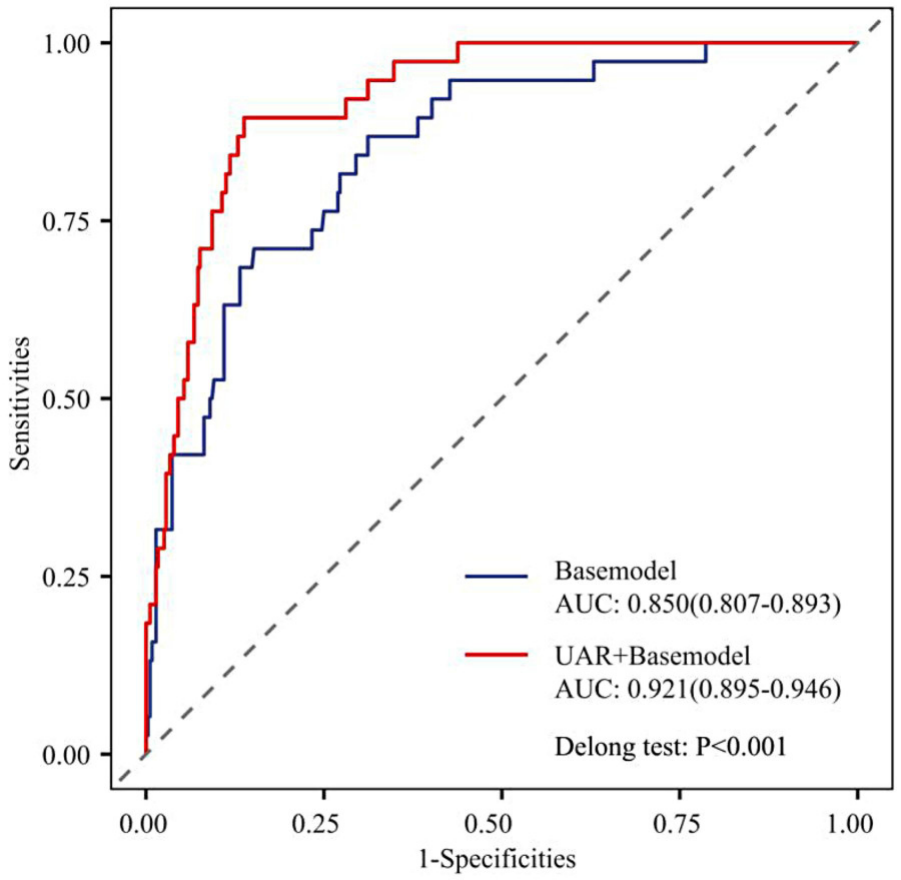
ROC curve comparison for predicting CIN after PCI in patients with coronary artery disease: basic model versus UAR + basic model.

The addition of UAR to the baseline prediction model significantly improved the predictive performance for CIN. The NRI was 0.296 (95% CI: 0.162–0.430) for categorical analysis and 0.797 (95% CI: 0.571–1.023) for continuous analysis. The IDI was 0.120 (95% CI: 0.063–0.178) (Table 3). Moreover, the AUC increased significantly from 0.850 for the baseline model to 0.921 after incorporating UAR (*P* < 0.001) (Figure 3). These results demonstrate that UAR provides substantial incremental value in enhancing the accuracy of CIN prediction.

**Table 3:** Incremental value of UAR in predicting CIN after PCI in patients with coronary artery disease.

Metrics	Base model	UAR + base model	*P*
NRI (Categorical)	ref	0.296 (0.162, 0.430)	<0.001
NRI (Continuous)	ref	0.797 (0.571, 1.023)	<0.001
IDI	ref	0.120 (0.063, 0.178)	<0.001
AUC	0.850	0.921	<0.001

Base model: DM + diuretic + heart rate + LVEF.

### Model development

**Figure 4:**
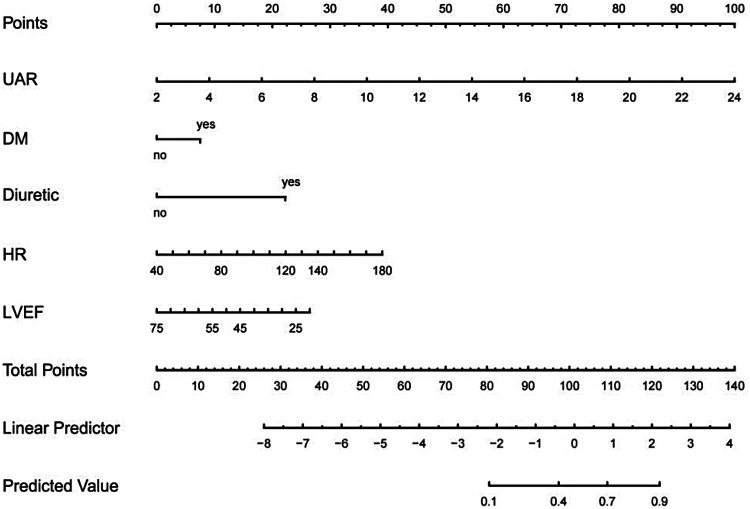
Nomogram for predicting CIN after PCI in patients with coronary artery disease.

We constructed a nomogram to quantify the individual risk of CIN after elective PCI (Figure 4). The model incorporates five clinical variables: DM, diuretic use, heart rate, LVEF, and UAR. By summing the points assigned to each variable, the probability of CIN can be estimated (range 10%–90%).

### Model validation

**Figure 5:**
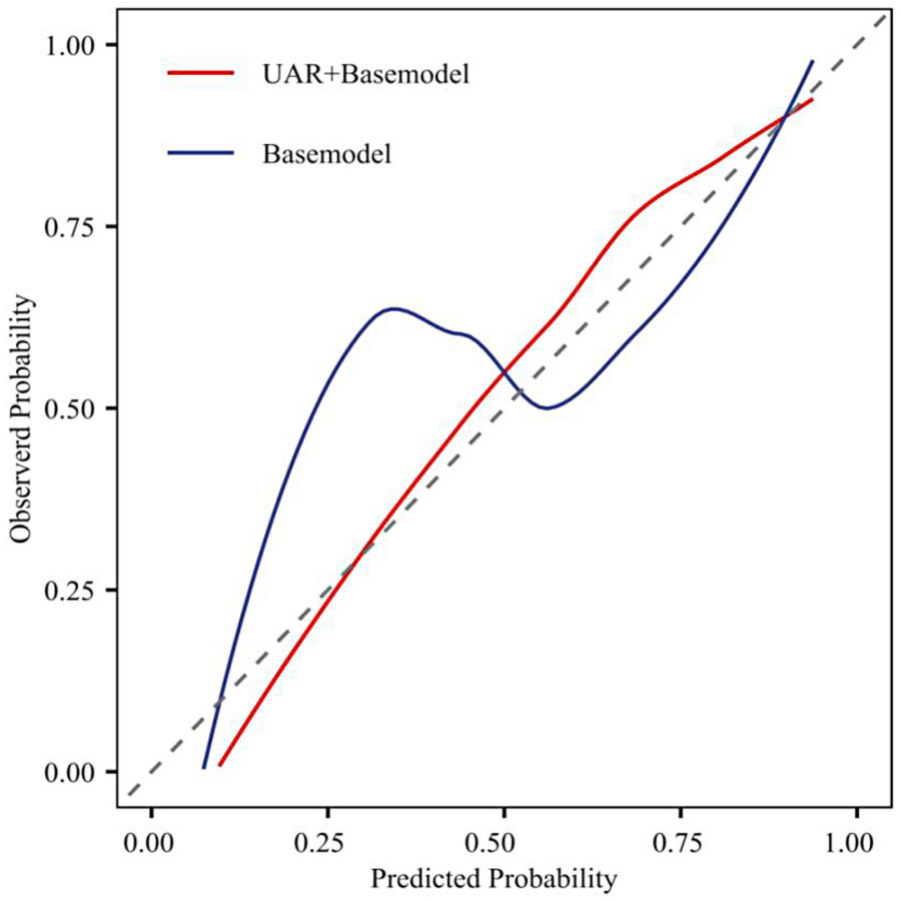
Calibration curve comparison for CIN after PCI in patients with coronary artery disease: basic model versus UAR + basic model.

**Figure 6:**
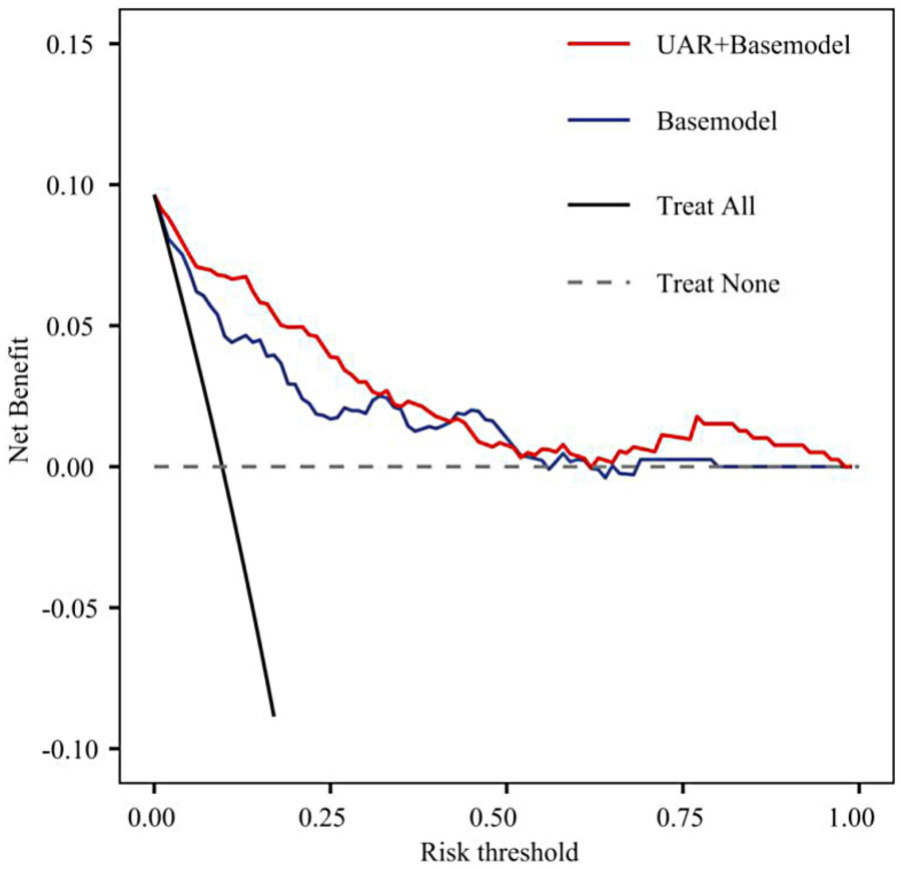
Clinical decision curve comparison for CIN after PCI in patients with coronary artery disease: basic model versus UAR + basic model.

Calibration curves demonstrated excellent agreement between predicted and observed probabilities across the entire risk spectrum for the UAR-enhanced model, with points lying close to the ideal 45° line (Figure 5). Decision-curve analysis showed that, across most clinically relevant threshold probabilities, the UAR-enhanced model yielded the highest net benefit, outperforming both the base model and the “treat-all” strategy (Figure 6). These findings confirm that incorporating UAR improves model calibration and optimizes clinical decision-making for CIN prevention.

## Discussion

In recent years, the prevalence of coronary artery disease has risen markedly as a consequence of an accelerated pace of life and lifestyle shifts. Although PCI is an effective and relatively safe treatment for complex cardiac conditions, prevention of CIN and identification of its high-risk determinants remain active research priorities. Focusing on an elective PCI cohort with coronary artery disease, we screened variables considered clinically relevant to CIN development. Univariate and multivariate logistic regression demonstrated that the UAR, DM, heart rate, LVEF, and diuretic use were independent predictors of CIN. A base model (incorporating all independent factors except UAR) and a UAR-enhanced model were subsequently constructed to quantify the incremental value of UAR. Reclassification and discrimination analyses revealed that adding UAR significantly improved the model's ability to identify incident CIN. ROC, decision-curve, and calibration plots consistently showed superior performance of the UAR-enhanced model. Finally, a nomogram was developed to visualise the logistic regression results, facilitating bedside application and dissemination. This easy-to-use tool should enable early recognition of high-risk individuals and inform tailored preventive strategies, thereby offering substantial clinical value for reducing CIN after PCI.

We identified UAR, diabetes mellitus, admission heart rate, LVEF, and diuretic use as independent determinants of CIN. Uric acid can precipitate within renal tubules, or extrarenal sites, whereas hypoalbuminaemia reflects chronic inflammation, protein-energy wasting, or impaired hepatic synthesis, conditions that collectively reduce renal reserve (41, 42). Persistent hyperglycaemia induces glomerular hyperfiltration, leading to a loss of functioning nephrons and diminished renal capacity (43, 44). Elevated heart rate signifies heightened sympathetic activity, which provokes renal vasoconstriction and decreases medullary perfusion, thereby amplifying the risk of CIN (45). Reduced LVEF indicates low cardiac output and renal underperfusion, triggering over-activation of the sympathetic and renin–angiotensin systems with increased inflammatory cytokines and reactive oxygen species, further predisposing to CIN (46). Previous studies have demonstrated that diuretics do not prevent CIN; instead, they may compromise systemic blood volume and renal perfusion, resulting in a decline in glomerular filtration rate that precipitates acute kidney injury (47). These findings underscore the need for careful evaluation and, when feasible, dose adjustment of diuretics before contrast administration to minimise CIN incidence.

In multivariable logistic regression, we did not identify contrast volume as an independent significant predictor of CIN, a finding consistent with previous studies (48). This observation may be explained by two factors. First, the contrast volume used in our cohort was relatively low. The mean contrast dosage in our study was 70.03 mL. According to the ESC/EACTS Guidelines (2014), to prevent CIN, the recommended targets for contrast administration are a volume <350 mL or <4 mL/kg body weight, or a V/CrCl ratio <3.7:1 (9). Second, A large systematic review and meta-analysis encompassing over 100,000 patients demonstrated insufficient evidence to support a statistically significant increase in acute kidney injury attributable to iodinated contrast media in the general population. This study suggested that the CIN observed in earlier reports may partly reflect patients' intrinsic risk factors-such as baseline renal insufficiency-rather than direct nephrotoxicity of contrast agents (49).

### Study limitations

This study has several limitations. First, the relatively modest sample size may have constrained statistical power and the generalizability of our findings to broader populations. Second, the analysis was based on a single-centre, retrospective design, lacking the robustness of prospective, randomized, controlled data. Third, body weight was not consistently recorded in our retrospective database, preventing the calculation of the contrast V/CrC, a validated predictor of CIN. Future prospective studies should incorporate this parameter to better quantify individualized contrast exposure.

## Conclusion

In patients with coronary artery disease undergoing elective PCI, the UAR is an independent predictor of CIN. Incorporating UAR significantly enhances the predictive power of the base model for CIN. We ultimately constructed a nomogram that visualizes the contribution of each variable to CIN. This easily calculated metric serves as a practical bedside tool for early identification of high-risk individuals, and the nomogram may guide the development of personalized prevention strategies to reduce the burden of complications after PCI and improve postoperative outcomes.

## Data Availability

The raw data supporting the conclusions of this article will be made available by the authors, without undue reservation.
